# Investigating the Anticonvulsant Properties of Aqueous Ethanolic Extracts of the Leaves, Roots, and Fruits of *Jatropha gossypifolia* L. (Euphorbiaceae)

**DOI:** 10.1155/2021/5547353

**Published:** 2021-06-11

**Authors:** Gideon Drafor, Emmanuel Duah, Nelson A. Ankamah, Godsway E. Kpene, Priscilla K. Mante

**Affiliations:** ^1^Department of Pharmacology, Faculty of Pharmacy and Pharmaceutical Sciences, College of Health Sciences, Kwame Nkrumah University of Science and Technology, Kumasi, Ghana; ^2^Department of Medical Laboratory Sciences, School of Allied Health Sciences, University of Health and Allied Sciences, Ho, Ghana

## Abstract

Convulsion is a typical symptom associated with epilepsy. *Jatropha gossypifolia*, a common plant in Ghana, has been used traditionally for the management of epilepsy. This study was carried out to ascertain the scientific basis for the traditional utility of *Jatropha gossypifolia* for various convulsive disorders and also determine the part of the plant with the most anticonvulsant activity. The anticonvulsant activity of the leaf, root, and fruit extracts in doses of 30–300 mg/kg was assessed using the picrotoxin-induced seizure models in mice. The drugs and chemical preparations used included diazepam, picrotoxin, ethanol (70%), and normal saline. GraphPad Prism 6 was used for all statistical analysis and plotting of graphs. Data were analyzed using one-way ANOVA, followed by Bonferroni's multiple comparison test. The leaf extract significantly and dose-independently reduced the frequency of myoclonic jerks (*P*=0.0001) and decreased the duration of clonic convulsions (*P*=0.019). The root extract also significantly and dose-dependently reduced the frequency of myoclonic jerks (*P*=0.001) but only decreased the frequency of tonic convulsions at 100 mg/kg (*P*=0.006). It also significantly decreased the duration of tonic convulsions (*P*=0.0001). The fruit extract only significantly and dose-independently reduced the frequency of myoclonic jerks (*P*=0.0001). It, however, showed an increase in the duration of both clonic and tonic convulsions. The study shows that the leaves and roots of *Jatropha gossypifolia* produce anticonvulsant activity which may be through enhancement of GABAergic transmission or activation of GABA receptors which support the traditional use of the plant to treat epileptic fits.

## 1. Introduction

Epilepsy is a disorder characterized by spontaneous recurring seizures that may arise from a focal area of the brain or diffusely resulting in various seizures culminating in episodic neuronal discharge [1, 2]. When elicited in a normal brain, by treatments such as electric shock or chemical convulsants without any incitement, a seizure is described as “nonepileptic” [3]. New-onset seizures have been found to occur more frequently among children younger than a year and adults above 55 years [4]. It is sad to note that an estimated 80–90% of epileptic patients in developing countries do not receive adequate treatment [5]. More worrying is the fact that a larger proportion of this group does not consult the medical doctor (MD) as first intention [5]. Invariably, a report by Amoateng et al. confirmed a similar situation in Ghana [6]. It was observed that pharmacological agents currently in clinical use inhibit seizures, but it is uncertain whether or not they can prevent the development of epilepsy [3]. Newer antiepileptic drugs (AEDs) such as lacosamide, lamotrigine, levetiracetam, oxcarbazepine, pregabalin, topiramate, and zonisamide are available, but the cost and adverse effects associated make them unattractive for most people especially the rural dwellers [7]. This negative economic impact of epilepsy does not only affect patients and family but also the community at large [8]. The most devastating of this burden is the stigma associated with the condition [9]. It has been found that stigma is positively associated with incomplete seizure control and poor psychosocial outcomes for people with epilepsy (PWE) [10].

In Africa and precisely in Ghana, traditional medicine plays a significant role in the management of various convulsive disorders [11]. A common plant used locally is *J. gossypifolia* (Linn). Its fruit is three-celled having one seed per cell, usually planted around houses and known for some therapeutic qualities [12, 13]. The leaves are used for intermittent fevers, carbuncles, eczema, itches, and sores on the tongues of babies, swollen mammae, stomach ache, and venereal disease [14]. The seeds possess emetic and purgative properties and thus are used for body pain [14, 15]. Besides, the stem latex of *J. gossypifolia* has been reported to be a haemostatic agent as well as having hepatoprotective potential [16, 17].

Although *Jatropha gossypifolia* is commonly used as an anticonvulsant in Ghana, there is a paucity of data on its anticonvulsant activity. In bridging this gap, there is a need to establish more robust scientific support for its traditional use. This study thus sought to demonstrate the anticonvulsant activity of the roots, leaves, and fruits of *Jatropha gossypifolia* (Euphorbiaceae).

## 2. Materials and Methods

### 2.1. Collection and Authentication of Plant Material

The leaves, roots, and fruits of *Jatropha gossypifolia* were collected and authenticated by the Department of Herbal Medicine (Voucher specimen numbers: KNUST/HM1/019/L093, KNUST/HM1/019/R023, and KNUST/HM1/019/F012) of the Faculty of Pharmacy and Pharmaceutical Sciences, Kwame Nkrumah University of Science and Technology (KNUST), Kumasi, Ghana. Plant collection was done at Aframso near Ejisu, a suburb of Ashanti region, Ghana.

### 2.2. Drugs and Chemicals

The drugs and chemicals used in this study included diazepam (Pharm-Inter, Brussels, Belgium), picrotoxin (Sigma-Aldrich Inc., St. Louis, MO, USA), ethanol (70%) (Brenntag Ghana Ltd.), and normal saline (0.9%). Purity of reagents was assessed via melting point determination and conformed to standards.

### 2.3. Ethics

The protocols for the study were approved by the Departmental Ethics Committees (D/COL/AE/R81).

### 2.4. Preparation of Aqueous Ethanolic Extract

Shade-dried leaves, roots, and sun-dried fruits of *Jatropha gossypifolia* were milled to a fine powder using a hammer mill. A quantity of 517 g ± 10 g of the finely powdered leaves, 300 g ± 10 g of the coarsely powdered roots, and 100 g ± 10 g of the coarsely powdered fruits were mixed with 2 l, 2.5 l, and 700 ml of 70% ethanol, respectively, and macerated for 3 days. The supernatant was filtered and concentrated by rotary evaporation at 78°C. The concentrate was completely evaporated in a hot air oven at a temperature of 50°C to obtain a gummy mass [18]. The resultant gummy brown solid extract was then stored in a well-labelled container at room temperature. The solutions of the leaf, root, and fruit extracts prepared in different concentrations using distilled water for analysis were named and labelled appropriately as JAGOL, JAGOR, and JAGOF, respectively. Percentage yields obtained for JAGOL, JAGOR, and JAGOF were 23.71% w/w, 28.12% w/w, and 20.93% w/w, respectively.

### 2.5. Experimental Animals

The animals used for this investigation were ICR mice. The animals weighed averagely 17–19 g. The animals were obtained from and kept in the Department of Pharmacology Animal House, KNUST where ambient environmental conditions of temperature, humidity, and light were provided. Physiological state of the animals was also ensured by the way of acclimatization before the experiment. Ethical standards were observed in treating the laboratory animals following standard laboratory animal care and use principles [19].

### 2.6. Picrotoxin-Induced Seizure Model

Male mice were divided into 13 groups, each with five members each (*n* = 5). The leaf, root, and fruit extracts (30, 100, and 300 mg/kg, *p.o*.) [20, 21] were administered to 9 groups while diazepam (0.1, 0.3, and 1.0 mg/kg, *i.p.*) was given to three other groups and the last group administered normal saline *i.p.* to serve as the control. After 1 hour and 30 minutes of treatment with drugs orally and intraperitoneally, respectively, each mouse was administered picrotoxin, 8 mg/kg subcutaneously [22]. The animals were placed individually in clear plastic observation chambers (3.5 cm × 3.5 cm × 3.5 cm) placed on a large plain glass covered with a brown paper elevated above the floor (60 cm). A digital video camera was positioned above the setup to videotape test sessions for 30 min. The Behaviour Tracker Software (JWatcher) version 9.0 was used to analyze the videos for the latency to the first myoclonic jerks, the latency to tonic convulsions, and the frequency as well as the duration of tonic and myoclonic convulsions for each mouse. Behaviours were manually scored using preset parameters in the tracker. Scoring was done independently by three authors (GD, ED, and NAA) in a blinded fashion and mean scores were further analyzed.

### 2.7. Phytochemical Analysis of the Ethanolic Extract

*Jatropha gossypifolia* was tested for the presence of tannins, glycosides, alkaloids, flavonoids, and triterpenoids by qualitative means.

#### 2.7.1. General Test for Tannins

0.5 g of the extract was boiled with 25 ml of water for 5 minutes, cooled, and filtered, and the volume was adjusted to 25 ml. To 1 ml of the extract, 10 ml of water was added, followed by the addition of 2 drops of 1% ferric chloride solution. It was then observed for precipitate formation. A blue-black precipitate observed in drops for hydrolyzable tannins while a dark green precipitate in excess indicated the presence of condensed tannins [23].

#### 2.7.2. General Test for Glycosides

200 mg of the extract was warmed with 5 ml of dilute sulphuric acid on a water bath for 2 minutes and filtered. The filtrate was made distinctly alkaline by adding four drops of 20% NaOH solution (confirmed with litmus paper). 1 ml each of Fehling's solutions A (a solution containing copper (II) sulphate, which is blue) and B (a clear liquid consisting of potassium sodium tartrate (Rochelle salt) and strong alkali, usually sodium hydroxide) was added to the filtrate and warmed on a water bath for about 2 minutes, and the colour of the precipitate formed was noted [23].

#### 2.7.3. General Test for Alkaloids

A small quantity of the plant extract was dissolved in distilled water. Few drops of Mayer's reagent were added. Formation of precipitates indicates a positive test for alkaloids [23].

#### 2.7.4. Flavonoids

A small quantity of the extract was macerated with distilled water and filtered into a flask. A strip of white filter paper was dipped into the filtrate, dried, and exposed to ammonia for 30 seconds to observe a colour change. The filter paper was then exposed to fumes of hydrochloric acid (HCl) until the disappearance of the colour change was observed [23].

#### 2.7.5. Triterpenoids

The extract was mixed with chloroform to obtain a chloroformic extract. Concentrated sulphuric acid was added to 5 ml of the extract carefully down the side of the test tube to form a layer. A reddish-brown colouration of the interface showed a positive result for the presence of triterpenoids [24].

### 2.8. Data Analysis

GraphPad Prism for Windows 6 (GraphPad Software, San Diego, CA, USA) was used for all statistical analysis and plotting of graphs. All data were analyzed using one-way ANOVA, followed by Bonferroni's multiple comparison test. All data passed the test for normality using the Shapiro–Wilk Test.

## 3. Results and Discussion

### 3.1. Phytochemical Analysis

JAGOL was found to contain secondary metabolites such as flavonoids, tannins, and cyclic triterpenes. JAGOR was found to have secondary metabolites such as alkaloids, tannins, and triterpenoids. JAGOF was found to contain flavonoids and tannins (Table 1).

### 3.2. Effects of Extracts on Picrotoxin-Induced Seizures

#### 3.2.1. Leaf Extract

**Figure 1:**
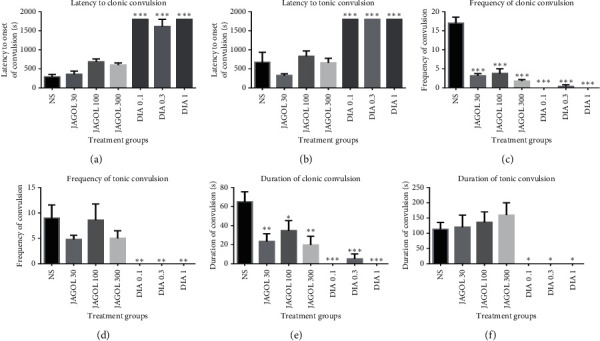
Effect of JAGOL (30–300 mg/kg, *p.o*.) and diazepam (0.1–1.0 mg/kg, *i.p*.) on the latency to PTX-induced (a) myoclonic and (b) tonic convulsions, frequency of (c) myoclonic and (d) tonic convulsions, and duration of (e) myoclonic and (f) tonic convulsions. Each column represents the mean ± SEM (*n* = 5). ^*∗∗∗*^*P* < 0.001, ^*∗∗*^*P* < 0.01, and ^*∗*^*P* < 0.05, one-way ANOVA followed by Bonferroni's multiple comparison test. JAGOL: *Jatropha gossypifolia* leaf extract; DIA: diazepam.

The leaf extract showed significant anticonvulsant activity against PTX-induced seizures. It increased the onset to PTX-induced myoclonic jerks though not statistically significant (*P*=0.304; Figure 1(a)). It significantly and dose-independently reduced the frequency of myoclonic jerks (*P*=0.0001; Figure 1(c)). Reduction in the frequency of myoclonic jerks by the extract was profound at all the doses used (*P*=0.0001 at 30–300 mg/kg). Again, it significantly decreased the duration of clonic convulsions in a dose-independent manner (*P*=0.019 at 30 and 300 mg/kg and *P*=0.033 at 100 mg/kg; Figure 1(e)).

Diazepam, an anticonvulsant, produced effects against PTX-induced seizures and the effects were dose-independent. The drug significantly delayed the onset of myoclonic jerks as well as the onset of tonic convulsions (*P*=0.0001; Figures 1(a) and 1(b), respectively). Also, diazepam caused a significant reduction in the frequency (*P*=0.0001, Figure 1(c); *P*=0.008, Figure 1(d)) and duration (*P*=0.0001, Figure 1(e); *P*=0.023, Figure 1(f)) of both myoclonic and tonic convulsions.

#### 3.2.2. Root Extract

**Figure 2:**
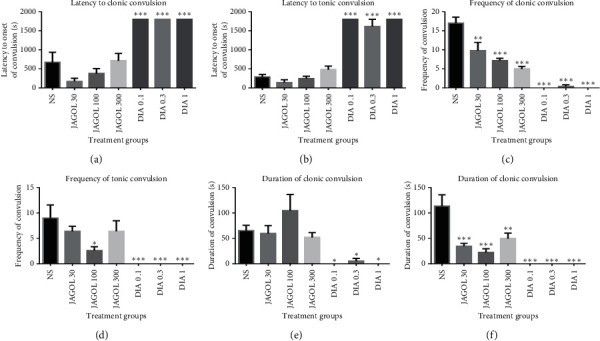
Effect of JAGOR (30–300 mg/kg, *p.o*.) and diazepam (0.1–1.0 mg/kg, *i.p*.) on the latency to PTX-induced (a) myoclonic and (b) tonic convulsions, frequency of (c) myoclonic and (d) tonic convulsions, and duration of (e) myoclonic and (f) tonic convulsions. Each column represents the mean ± SEM (*n* = 5). ^*∗∗∗*^*P* < 0.001, ^*∗∗*^*P* < 0.01, and ^*∗*^*P* < 0.05, one-way ANOVA followed by Bonferroni's multiple comparison test. JAGOR: *Jatropha gossypifolia* root extract; DIA: diazepam.

The root extract showed significant anticonvulsant activity against PTX-induced seizures. However, JAGOR did not cause a significant delay in the latency to myoclonic jerks and tonic convulsions (*P*=0.981; Figures 2(a) and 2(b), respectively). It significantly and dose-dependently decreased the frequency of myoclonic jerks (*P*=0.008 at 30 mg/kg and *P*=0.001 at 100–300 mg/kg; Figure 2(c)) and also significantly decreased the frequency of tonic convulsions at 100 mg/kg (*P*=0.006; Figure 2(d)). Reduction in the duration of tonic convulsions by JAGOR was significant at all the doses used (*P*=0.0001 at 30–100 mg/kg and *P*=0.004 at 300 mg/kg; Figure 2(f)).

Also diazepam, an anticonvulsant, produced effects similar to that of JAGOR against PTX-induced seizures and the effects were dose-independent. The drug significantly delayed the onset of myoclonic jerks as well as the onset of tonic convulsions (*P*=0.0001; Figures 2(a) and 2(b)). Also, diazepam caused a significant reduction in the frequency and duration of myoclonic and tonic convulsions (*P*=0.0001, Figures 2(c), 2(d), and 2(f); *P*=0.027, Figure 2(e)).

#### 3.2.3. Fruit Extract

**Figure 3:**
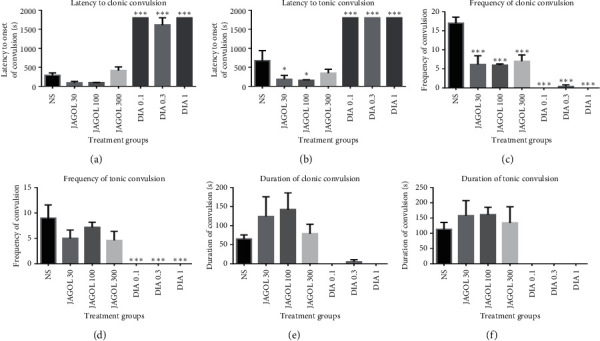
Effect of JAGOF (30–300 mg/kg, *p.o*.) and diazepam (0.1–1.0 mg/kg, *i.p*.) on the latency to PTX-induced (a) myoclonic and (b) tonic convulsions, frequency of (c) myoclonic and (d) tonic convulsions, and duration of (e) myoclonic and (f) tonic convulsions. Each column represents the mean ± SEM (*n* = 5). ^*∗∗∗*^*P* < 0.001 and ^*∗*^*P* < 0.05, one-way ANOVA followed by Bonferroni's multiple comparison test. JAGOF: *Jatropha gossypifolia* fruit extract; DIA: diazepam.

The fruit extracts significantly and dose-independently reduced the frequency of myoclonic jerks (*P*=0.0001; Figure 3(c)). Reduction in the frequency of myoclonic jerks by the extract was significant at all the doses used (*P*=0.0001). The extract, however, showed an increase in the duration of both clonic and tonic convulsions (Figures 3(e) and 3(f)).

Diazepam, an anticonvulsant, produced effects against PTX-induced seizures and the effects were dose-independent. The drug significantly delayed the onset of myoclonic jerks (*P*=0.0001; Figure 3(a)) as well as the onset of tonic convulsions (Figure 3(b)). Also, diazepam caused a significant reduction in the frequency of myoclonic and tonic convulsions (*P*=0.0001; Figures 3(c) and 3(d)).

## 4. Discussion

*J. gossypifolia* has been scarcely studied with regard to its anticonvulsant effects even though its use in this regard by the local folks is enormous. The outcome of studying the influence of *J*. *gossypifolia* on reversing picrotoxin-induced convulsion provide evidence that the aqueous ethanolic extract of the leaf and root of *J. gossypifolia* possesses anticonvulsant properties and may be of use in various forms of seizures.

The study showed the leaf and root extracts having a significant anticonvulsant activity against PTX-induced seizures by significantly reducing the frequency and duration of convulsions with the fruit extract showing only a significant reduction in the frequency of myoclonic jerks.

The mechanism of picrotoxin antagonism of ionotropic GABA receptors is still controversial. That notwithstanding, clear differences in affinity have been demonstrated by picrotoxin and other chloride channel blockers on GABA_A_ and GABA_C_ receptors [25, 26]. However, the effects exhibited on both receptors by picrotoxin have been found to be similar [27]. According to Newland and Cull-Candy [26], picrotoxin exerts a complex channel blocking mechanism on the GABA receptor by stabilizing a closed form of the receptor channel and or causing an allosteric block of the channel. Agents that enhance GABAergic neurotransmission have been shown to inhibit or attenuate seizures, while those that inhibit GABAergic activity advance and facilitate seizures [25].

Given that previous studies have revealed the analgesic action of *Jatropha gossypifolia* [27] with another study on locomotor activity affirming its CNS depressant activity [14], the effects of our extract on PTX-induced convulsions are not surprising. Since the leaf and root extracts both showed a remarkable dose-independent anticonvulsant activity against PTX-induced clonic seizures which is in line with its CNS depressant activity, our study has shown that a more appropriate option will be to promote the use of the leaf over the root to preserve the plant. Also, given that Abreu and colleagues have established the hypotensive and vasorelaxant effects of ethanolic extract from *Jatropha gossypifolia* L. in rats, our study shows promises of getting new leads from the plant, which will be effective in treating conditions in which hypertension and convulsion are both of concern such as in preeclampsia and eclampsia [20].

Flavonoids have been demonstrated to antagonize the responses mediated by GABA_A_ and GABA_C_ receptors [28]. Also, steroids have been shown to modulate ionotropic GABA receptors [29]. Furthermore, research on phytoconstituents and plants suggests the potential of flavonoids and steroids being ligands for the GABA_A_ receptor in the CNS, which led to the assertion that they can act as benzodiazepine-like molecules [30].

The outcome of the phytochemical analysis of the leaf extract, which showed the presence of flavonoids and steroids, is in line with the literature [14]. The presence of flavonoids and steroids also affirms its remarkable anticonvulsant activity compared to the root and fruit extracts. The activity of the root extract can, therefore, be attributed to the presence of steroids. Even though flavonoids were found to be present in the fruit extract, they produced a less remarkable anticonvulsant effect with only a significant reduction in the frequency of clonic convulsion.

These results point to a possible synergy shown by flavonoids and steroids present in the leaf extract. The outcome of the phytochemical analysis of the various plant parts, therefore, confirms the magnitude of activities exhibited in reversing PTX-induced seizures. The results of this study also corroborated literature which stated that the leaves and roots of *Jatropha gossypifolia* have been shown to be effective against convulsions, fever, and hypertension convulsions [31].

The standard anticonvulsant, diazepam, which was used to confirm the efficiency of the seizure model caused a significant delay to the onset of myoclonic jerks and tonic convulsions and reduced the frequency and duration of myoclonic and tonic convulsions induced by PTX as well. It is, therefore, likely that the extracts attenuated PTX convulsion by enhancing GABA neurotransmission. Also, an antioxidant action may contribute to anticonvulsant effects due to the presence of flavonoids in these extracts even though additional seizure models such as the pilocarpine-induced and pentylenetetrazole-induced seizure models may be needed to affirm these assertions [32, 33].

The remarkable inhibition against convulsions in the PTX model by the leaf and root extracts is suggestive of the fact that they may be enhancing GABAergic inhibition in the CNS or may be due to the activation of GABA receptor by the extracts.

### 4.1. Strengths and Limitations

This study provides valuable data that established that the leaves of *Jatropha gossypifolia* possess comparable anticonvulsant activity as the roots. This provides a basis for the use of the leaves in place of the commonly used roots; this will contribute largely to plant conservation efforts, traditionally. The study, however, could benefit from testing of the extracts in other acute or chronic models of epilepsy.

## 5. Conclusions

The outcome of the study of the aqueous ethanolic extract of the leaf, root, and fruit extracts of *Jatropha gossypifolia* indicated that the plant contains secondary metabolites that possess anticonvulsant effects with the leaf and root being more potent in this regard compared with the fruit. These data support the use of the leaves in place of the root to promote plant preservation. However, further work needs to be carried out on the roots and leaves using other acute and chronic models of epilepsy. Furthermore, additional neuropharmacological testing such as anxiolytic and analgesic properties of the roots and leaves should be carried out to ascertain the full neuropharmacological profile of these plant parts. Lastly, toxicological studies need to be done to establish the safety profile of this plant.

## Figures and Tables

**Table 1:** Phytochemical analysis of aqueous ethanolic extract of *Jatropha gossypifolia*.

Test	Result		
	Leaves	Roots	Fruits
Alkaloid	Absent	Present	Absent
Flavonoid	Present	Absent	Present
Tannins	Present	Present	Present
Tritepenoids	Present	Present	Absent

## Data Availability

The datasets used during the current study are available from the corresponding author upon request.
